# Has the COVID-19 Pandemic Affected Lay Beliefs about the Cause and Course of Mental Illness?

**DOI:** 10.3390/ijerph18094912

**Published:** 2021-05-05

**Authors:** Cliodhna O’Connor

**Affiliations:** School of Psychology, University College Dublin, Dublin 4, Ireland; cliodhna.oconnor1@ucd.ie

**Keywords:** anxiety, depression, COVID-19, pandemic, lay beliefs, attribution, illness perceptions, folk psychiatry

## Abstract

COVID-19 and its countermeasures have negatively impacted the mental health of populations worldwide. The current paper considers whether the rising incidence of psychiatric symptoms during the pandemic may affect lay beliefs about the cause and course of mental illness. Laypeople’s causal attributions and expectations regarding the trajectory of mental illness have important implications for societal stigma and therapeutic orientations. Two online experimental studies investigated whether reading about fictional cases of mental illness that were explicitly situated during the COVID-19 pandemic, compared with reading about the same cases without any pandemic-related contextualisation, affected attributions and expectations about Generalised Anxiety Disorder (Study 1) and Major Depressive Disorder (Study 2). Study 1 (*n* = 137) results showed that highlighting the onset of anxiety symptoms during the COVID-19 pandemic weakened attributions to biological causes and reduced the anticipated duration of symptoms. However, Study 2 (*n* = 129) revealed no effects of COVID-19 contextualisation on beliefs about the cause or course of depression. The research provides preliminary evidence that the increased incidence of mental illness during the pandemic may reshape public beliefs about certain mental illnesses. Given the importance of public understandings for the lived experience of mentally unwell persons in society, further evidence of the range and extent of the pandemic’s effects on lay beliefs is important to inform clinical, public health and stigma-reduction initiatives.

## 1. Introduction

Lay understandings of the cause and course of mental illness have important implications for stigma, help-seeking, and treatment selection and engagement [1,2]. Despite evidence that lay beliefs about mental illness shift across time [3], little research has explored how lay beliefs are affected by specific historical or societal events. COVID-19 and its countermeasures have significantly impacted the mental health of populations around the world, with a particularly steep increase in depression and anxiety symptoms [4,5,6,7]. The current paper reports two experimental studies that explore whether highlighting the onset of mental illness during the COVID-19 pandemic affects lay attributions or expectations about depression and anxiety symptoms.

The emergence of the novel coronavirus COVID-19, declared a pandemic in March 2020, had unprecedented global impacts in mortality, illness and societal disruption. In the absence of effective treatments or vaccines in the early stages of the pandemic, the only available measures to curtail the spread of the virus involved constraining public behaviour through travel restrictions, closure of non-essential services, and prohibition on cross-household contacts. By April 2020, half of the world’s population was subject to ‘lockdown’ or ‘stay-at-home’ orders [8].

These restrictions on social, recreational and economic activity, while necessary to contain a deadly virus, represented a further threat to populations’ health and wellbeing. Meaningful social connections are a key pillar of psychological wellbeing; conversely, social isolation is a major risk factor for mental ill-health [9,10]. It is likely that restricted opportunities for social engagement during the pandemic negatively impacted many people’s mental health [11]. This was compounded by other psychopathological risk factors that the pandemic amplified, including unemployment and under-employment, economic insecurity, inequalities related to gender, race and class, caring stress, and worry about the safety of self or others [12].

Research investigating the mental health impacts of COVID-19 and its countermeasures has identified a particularly sharp rise in depression and anxiety symptoms [4,5,6,7]. A meta-analysis of 12 studies undertaken during the pandemic reported a pooled prevalence of depression of 25%—a seven-fold increase on the estimated global prevalence of depression in 2017 (3.44%) [13]. Similarly, meta-analytic evidence yielded an estimated prevalence of clinical anxiety during the pandemic of 25%, which is triple the global prevalence of anxiety disorders prior to the pandemic [14]. One interpretation of these statistics is that they highlight the malleability of mental health and illness to environmental or situational factors [15]. The role of life events in the aetiology of depression and anxiety, independently or in conjunction with biological vulnerabilities, is well-established scientifically [16,17,18,19]. However, research shows that people have a propensity to underestimate the social determinants of health and illness [20].

In the mental health sphere, patterns of lay causal attribution for mental illness represent one dimension of ‘folk psychiatry’, a field of research that theoretically prioritises common-sense, rather than scientific, understandings of mental health and illness [21]. Folk psychiatric representations that undervalue the social causes of distress can be considered within the conceptual framework of ‘biological essentialism’ [22]. Essentialism is defined as a propensity to explain the observable characteristics of social categories in terms of a deeper underlying ‘essence’ shared by all category-members [23]. Essentialist representations of psychiatric categories are supported by attributing mental illness to biological causes, with brains, genes or hormones standing as placeholders for the defining ‘essence’ [24,25]. While essentialism is a common tendency in human cognition, it can be promoted or mitigated by socio-cultural contexts [26]. It is therefore possible that the increased rates of psychiatric symptoms observed during COVID-19 pandemic may reduce essentialism by shifting lay explanations of mental illness away from biological towards social, environmental or situational attributions.

The essentialism literature suggests that any such change in public attribution patterns is likely to affect the lived experiences of mentally unwell people in society. When lay populations attribute mental illness to primarily biological causes, this has a range of consequences for social responses to people with mental illness. For instance, research indicates that attributing mental illness to biological causes reduces blame of people with mental illness, but increases perceptions of dangerousness, social ostracism and fatalism about their prospects of recovery [2,27]. Moreover, at a societal level, public underestimation of the social determinants of distress may dampen support for mental health policies that focus on environmental interventions (e.g., community-level supports) rather than biomedical treatments (e.g., medication) [28,29,30]. This is likely to disadvantage populations who experience mental illness, given evidence of the effectiveness of socially-targeted interventions in promoting mental health and wellbeing [31].

Alongside influencing lay understandings of the root causes of mental illness, the COVID-19 pandemic may also affect another aspect of folk psychiatry: expectations of the future trajectory of mental health difficulties. According to the widely-used self-regulation model [32], beliefs about the course of an illness can be decomposed into the specific dimensions of its perceived treatability, susceptibility to personal control, and timeline or duration. It is possible that the COVID-19 pandemic may affect each of these dimensions. For instance, observing that mental illness symptoms arise in response to the pandemic could reduce the anticipated duration of the illness (if symptom remission is expected to coincide with the end of the pandemic), undermine confidence in the likelihood of attaining effective treatment (given widespread experiences of strained healthcare resources), or lessen the perceived personal controllability of symptoms (due to their contingency on large-scale global events). Any such changes to lay beliefs about the course of mental illness could, in turn, have implications for the ways that people at risk of mental health difficulties choose or are advised to manage their symptoms: research shows that perceiving mental illness symptoms as chronic, treatable, and controllable encourages help-seeking, active coping and treatment engagement [1]. To date, all such chains of effects remain speculative, as no research has empirically investigated whether or how the pandemic has affected anticipated trajectories of mental illness.

The current research explores whether the context of the COVID-19 pandemic has affected lay understandings of the causes and course of mental illness. A vignette-based experimental design, with a UK sample, is used to investigate whether reading about fictional cases of mental illness that are explicitly situated during the COVID-19 pandemic, compared with reading about the same cases without any pandemic-related contextualisation, affects attributions and expectations about the described symptoms. Informed by evidence that anxiety and depression are the psychological symptoms with the greatest increase during the pandemic [4,5,6,7], the paper presents two studies, which explore lay responses to fictional cases of (1) Generalised Anxiety Disorder (GAD) and (2) Major Depressive Disorder (MDD). The research aims to answer the following questions:

- Does highlighting the onset of mental illness symptoms during the COVID-19 pandemic affect people’s tendency to attribute those symptoms to biological or social causes?
- Does highlighting the onset of mental illness symptoms during the COVID-19 pandemic affect people’s expectations regarding the timeline, treatability or controllability of the symptoms?

## 2. Study 1: Generalised Anxiety Disorder

### 2.1. Materials and Methods

#### 2.1.1. Design

A between-groups experimental study was performed using a contrastive vignettes design [33]. Vignettes (Appendix A) were adapted from previous research [34]. Data were collected online using the Qualtrics platform. Data collection took place during early March 2021, when the COVID-19 pandemic was ongoing and government restrictions imposed in the UK, though with cases decreasing and the vaccination programme underway.

#### 2.1.2. Participants

A priori power analysis indicated a sample size of *n* = 128 was required to detect a medium effect with alpha of 5% and power of 80%. Participants were recruited through the crowdsourcing platform Prolific, with participants paid £1GBP for participation in research advertised as a study of “beliefs and attitudes about mental illness”. Pre-study screening criteria specified that all participants should live in the UK, speak fluent English, be aged between 18–65 years, and not have been previously diagnosed with a mood or anxiety disorder.

Captcha and attention-checks were embedded within the survey to exclude ‘bots’ or inattentive participants. Exclusion of responses that failed such validation measures left 137 participants. Of these, 70.1% (*n* = 96) were female and 79.6% (*n* = 109) identified their ethnicity as White/Caucasian. Two-thirds (66.5%, *n* = 91) were in paid employment, 15.3% (*n* = 21) were students and the remainder were unemployed or retired. University-level education was reported by 64.2% (*n* = 88). Under half (46.7%, *n* = 64) reported personally knowing someone who had received a diagnosis of GAD; 35.0% (*n* = 48) identified this person as a family member or close friend.

#### 2.1.3. Procedure

After consenting to participate by ticking a box at the start of the online study, participants were randomly assigned to read one of two vignettes. Vignettes (Appendix A) described an identical case of a non-gendered young person (‘Alex’) with the symptoms and diagnosis of GAD, according to ICD-10 diagnostic criteria. In the COVID-19 condition, participants read that the symptoms experienced by this 25-year-old had begun during the COVID-19 pandemic. The Control vignette did not mention the pandemic, instead simply stating that the symptoms began when Alex was aged 25.

After reading the vignettes, participants completed an attention check that confirmed accurate recall of details from the vignette. They then completed two validated measures:

- Attribution of GAD symptoms to Biological/Heredity and Social/Environmental causes was measured using two subscales of the Mental Illness Attribution Questionnaire [35]. This measure has been validated in international samples with documented internal consistency, convergent validity, and test-retest reliability [35]. On a 7-point Likert scale, participants rated the likelihood that a range of factors caused the types of mental health difficulties described in the vignette. The measure includes 17 items assessing attributions to Social/Environmental causes (e.g., ‘Stressful life circumstances’, ‘Loneliness’, α = 0.95), and seven items assessing Biological/Heredity attributions (e.g., ‘Chemical imbalance in the brain’, ‘Genes or heredity’, α = 0.81). The composite items for each subscale were averaged, making the range for each subscale 1–7, with higher scores indicating greater endorsement of Social/Environmental or Biological/Heredity causes. Items were presented to participants in randomised order.
- Beliefs about the course of Alex’s illness were measured by adapting three subscales from the Revised Illness Perceptions Questionnaire [36]. This measure can be applied to study perceptions of a range of different illnesses, with established internal and test-retest reliability, and discriminant, known group and predictive validity [36]. The Timeline subscale contained four items (α = 0.73) measuring perceived length of Alex’s illness (e.g., ‘Alex’s problem will last for a long time’); average higher scores represent expectations symptoms were chronic rather than acute. The four items in the Treatability subscale (α = 0.76) assessed perceptions that symptoms were amenable to treatment (e.g., ‘Treatment will be effective in resolving Alex’s problem’); average higher scores indicate stronger beliefs in treatability. Four items assessed perceptions of Personal Control (α = 0.67) over the illness (e.g., ‘Alex has the power to influence the problem’); average higher scores indicate higher perceived individual control. All responses were provided on a 7-point Likert scale of agreement, with items presented in randomised order. Four items were reverse-scored in accordance with scale instructions.

The questionnaire concluded with a battery of socio-demographic questions, followed by a debriefing page. Automated reminders of unanswered questions were embedded throughout to minimise missing data.

#### 2.1.4. Analysis

Data were analysed using SPSS v24 (IBM Corp, Armonk, NY, USA). Preliminary analysis investigated the equivalence of socio-demographic profiles across experimental groups and the relationship between socio-demographic and dependent variables. Results of these analyses informed the selection of covariates in subsequent ANCOVA tests of the effect of experimental condition on dependent variables. Only one case had missing data (<5% of items incomplete) and was excluded pairwise. As the research was exploratory, all hypotheses were two-sided and the analysis did not correct for multiple comparisons, but included measures of effect size.

### 2.2. Results

#### 2.2.1. Preliminary Analyses

The survey software randomly assigned 68 participants to the COVID-19 condition and 69 to the Control condition. Preliminary analyses (chi-square/Pearson’s correlation tests) revealed no cross-condition differences in the distribution of gender, age, ethnicity, or personal acquaintance with someone with GAD (all *p* > 0.05). However, there were more university-educated people in the COVID-19 (75.0%) than Control (53.6%) condition, χ^2^(1, 137) = 6.812, *p* = 0.009.

Exploratory tests of the relationship between socio-demographic and dependent variables revealed no significant effects of age, ethnicity, or personal acquaintance with someone with GAD (all *p* > 0.05). However, university educated participants (*M* = 4.22, *SD* = 0.88) believed that the symptoms would have a shorter Timeline than non-university participants (*M* = 4.57, *SD* = 0.86), *t*(135) = 2.23, *p* = 0.03. Women (*M* = 5.59, *SD* = 0.81) saw the symptoms as having higher Treatability than men (*M* = 5.19, *SD* = 0.81), *t*(135) = 2.62, *p* = 0.01. Women (*M* = 4.89, *SD* = 1.25) made stronger attributions to Social/Environmental causes than men (*M* = 4.30, *SD* = 1.26), *t*(135) = 2.52, *p* = 0.01. Women (*M* = 4.06, *SD* = 1.19) also reported higher scores than men (*M* = 3.52, *SD* = 1.28) for Biological/Heredity causes, *t*(134) = 2.38, *p* = 0.02. 

Descriptive statistics are presented in Table 1.

#### 2.2.2. Effects of Experimental Manipulation

Based on the above preliminary results, gender and education were included as covariates in testing the effect of the experimental manipulation. Results showed that (controlling for gender and education) the Control condition made significantly stronger attributions to Biological/Heredity causes than the COVID-19 condition, *F*(1,132) = 6.38, *p* = 0.01, η_p_^2^ = 0.05. There was no significant effect of experimental condition on Social/Environmental causes, *F*(1,133) = 1.35, *p* = 0.25, η_p_^2^ = 0.01.

Results revealed that the experimental manipulation did not affect perceptions of Treatability, *F*(1,133) = 1.59, *p* = 0.21, η_p_^2^ = 0.01, or Personal Control, *F*(1,133) = 1.70, *p* = 0.20, η_p_^2^ = 0.01. However, participants in the Control condition expected a significantly longer Timeline than in the COVID-19 condition, *F*(1,133) = 6.60, *p* = 0.01, η_p_^2^ = 0.05.

#### 2.2.3. Summary of Study 1 Results

The results of Study 1 suggested that highlighting the COVID-19 context for a case of GAD weakened attributions to biological causes and reduced the anticipated duration of symptoms.

## 3. Study 2: Major Depressive Disorder

### 3.1. Materials and Methods

#### 3.1.1. Design

The study design was identical to Study 1, but vignettes (Appendix B) described a case of MDD.

#### 3.1.2. Participants

As for Study 1, participants were recruited via Prolific. However, people who had completed Study 1 were unable to complete Study 2. After exclusion of those who failed attention checks, there were 129 participants. The majority were female (66.7%, *n* = 86) and identified as White/Caucasian (82.2%, *n* = 106). Most were in paid employment (67.4%, *n* = 87) or students (19.4%, *n* = 25). Nearly two-thirds (65.9%, *n* = 85) had been educated to university-level. A large minority (40.3%, *n* = 52) reported personally knowing someone who had received a diagnosis of MDD; 27.9% (*n* = 36) identified this person as a family member or close friend.

#### 3.1.3. Procedure

The study proceeded in a manner identical to Study 1. 

#### 3.1.4. Analysis

The same analytic procedures were used as for Study 1. There were no missing data.

### 3.2. Results

#### 3.2.1. Preliminary Analyses

The COVID-19 condition was assigned 65 participants and the Control condition 64 participants. Preliminary checks indicated the conditions were equivalent in the distribution of gender, education, ethnicity, and personal acquaintance with someone with MDD (all *p* > 0.05). However, people in the COVID-19 condition (*M* = 38.35, *SD* = 12.70) were significantly older than Control participants (*M* = 30.91, *SD* = 10.30), *t*(127) = 3.65, *p* < 0.001.

Perceptions of the cause or course of symptoms were not significantly related to gender, education, ethnicity, or personal acquaintance with someone with MDD (all *p* > 0.05). However, age was significantly correlated with Treatability beliefs, *r*(129) = 0.278, *p* = 0.001.

Table 2 presents descriptive statistics.

#### 3.2.2. Effects of Experimental Manipulation

Based on the above preliminary results, age was included as a covariate in testing the effect of the experimental manipulation. As in Study 1, participants in the Control condition showed stronger attributions to Biological/Heredity causes than the COVID-19 condition; however, this difference was not significant, *F*(1,126) = 2.24, *p* = 0.14, η_p_^2^ = 0.02. Neither was there a significant effect of experimental condition on attribution to Social/Environmental causes, *F*(1,126) = 0.16, *p* = 0.69, η_p_^2^ < 0.01.

Results revealed no effect of experimental manipulation on perceptions of Treatability, *F*(1,126) = 0.53, *p* = 0.47, η_p_^2^ < 0.01, Personal Control, *F*(1,126) = 0.51, *p* = 0.48, η_p_^2^ < 0.01, or Timeline, *F*(1,126) = 1.66, *p* = 0.20, η_p_^2^ = 0.01.

#### 3.2.3. Summary of Study 2 Results

Results revealed that, deviating from Study 1′s findings for GAD, highlighting the COVID-19 context for a case of MDD did not affect lay beliefs about the cause or course of depressive symptoms.

## 4. Discussion

Folk psychiatric beliefs about the cause and course of mental illness may affect the lived experiences of people with mental illness by modulating levels of societal stigma, therapeutic engagement, and public support for different policy approaches. This research offers a first investigation of how the COVID-19 pandemic may affect lay beliefs about mental illness. It suggests that observing the rise of anxiety disorders during the pandemic may shift lay understandings by undermining the popularity of biological attributions, and by reducing the anticipated duration of anxiety symptoms. However, such effects may be specific to GAD; although rates of depression have also seen a marked increase during the pandemic [4,5,6,7], highlighting this context for a case of MDD did not affect lay beliefs about depression’s cause or course.

While results showed that making salient the COVID-19 context affected causal attributions for anxiety symptoms, it is interesting that this effect was observed in biological rather than social attributions. Despite the logical inference that rising rates of mental illness during the pandemic reveal the environmental contingency of mental health, mere awareness of the pandemic-related context for a case of GAD did not specifically sensitise people to the situational determinants of anxiety. This may be due to a ceiling effect; in both studies and across conditions, agreement with social/environmental causal attributions was consistently high and preferred over biological/heredity attributions. Instead, Study 1 results showed that COVID-19 contextualisation reduced endorsement of biological attributions for GAD. The likely implications of this finding are ambiguous, due to the ‘mixed blessings’ typically attached to biological attributions for mental illness [37]. While biological attributions produce a range of both positive and negative social responses (e.g., reducing blame but increasing social distance and stereotyping), their net effects for the social experiences of people with mental illness appear more negative than positive [2,27]. It is therefore possible that in the general population, observing the links between anxiety and the COVID-19 pandemic will reduce biological essentialism, which may help ameliorate the stigmatisation of those with anxiety disorders.

Similar ambiguities surround interpretation of the finding that COVID-19 contextualisation reduced the anticipated duration of anxiety symptoms. In people experiencing mental illness, belief that one’s illness is chronic in duration is associated with worse symptom severity, but increased help-seeking and active coping [1]. If the general public expects that anxiety disorders that arise during the COVID-19 pandemic will be of shorter duration, this could reflect optimism regarding unwell persons’ ability to regain a state of wellbeing. Alternatively, anticipating rapid remission of symptoms might reflect minimisation of their severity and impede help-seeking in people who do experience anxiety symptoms. Further research is necessary to clarify the wider repercussions of a shorter perceived duration of symptoms.

One contribution of this research is to highlight that the effects of the COVID-19 pandemic on mental illness beliefs may differ depending on the specific disorder in question. This aligns with emerging evidence that public attitudes to different psychiatric categories are sustained by unique and divergent networks of beliefs [38,39,40]. While highlighting the onset of symptoms during the pandemic affected lay beliefs about GAD, beliefs about MDD were not mutable to this experimental manipulation. This may be because the causal links between the pandemic and anxiety seem more direct, with anxiety a natural emotional response to facing a threat to one’s physical welfare. Alternatively, given evidence that public awareness of depression is higher than that of anxiety disorders [41,42], perhaps folk psychiatric views of depression are more engrained and less malleable to intervention. Further research is required to confirm that lay beliefs about depression are indeed unaffected by its increased incidence during the pandemic, and to expand investigation to other psychiatric categories beyond mood and anxiety disorders.

Future research would also benefit from including a wider variety of outcome variables. Interpretation of the results obtained in the current studies would have been aided by additional measures of stigmatising attitudes and behavioural responses to people with mental illness. Further, notwithstanding the random assignment to experimental conditions, inclusion of pre-post measures would have helped confirm the unique effects of the experimental manipulation. The studies’ samples were limited to UK clients of an online recruitment platform: while such platforms typically produce superior samples to convenience sampling methods [43,44,45], study samples were biased towards female, white and university-educated participants. The studies’ scope is also limited by delivering the experimental manipulation through text vignettes. While vignettes are a popular approach to study attitudes to mental illness [46], offering the potential for controlled yet ecologically valid experimental stimuli, their reliance on specific fictional cases impedes the generalisability of results. In the current research, it is unclear whether responses to these specific narratives of anxiety and depression symptoms generalise to indicate enduring effects on public beliefs about the disorder categories of GAD or MDD. Further longitudinal research is needed to track the long-term implications of the pandemic for attitudes and beliefs regarding mental illness.

## 5. Conclusions

At the time of writing, there are grounds for optimism that the emergence of effective vaccines will expedite the end of the COVID-19 pandemic. Yet even after the virus has been contained, societies around the world will be obliged to reckon with the legacy it leaves behind. Some have voiced concern that the decline of the COVID-19 pandemic will be followed by a ‘second pandemic’ of mental illness due to distress caused, exacerbated or untreated during the pandemic [47]. The current research provides preliminary evidence that the heightened incidence of mental illness during the pandemic may reshape public beliefs about certain mental illnesses. Given the importance of public understandings for the lived experience of mentally unwell persons in society, evidence of the range and extent of the pandemic’s effects on lay beliefs is important to inform clinical, public health and stigma-reduction initiatives.

## Figures and Tables

**Table 1:** Descriptive statistics across experimental conditions in Study 1.

Variable	COVID-19 Condition Mean (*SD*)	Control Condition Mean (*SD*)
Social/Environmental Causes	4.64 (1.42)	4.78 (1.13)
Biological/Heredity Causes	3.66 (1.23)	4.14 (1.22)
Timeline	4.13 (0.89)	4.56 (0.84)
Treatability	5.39 (0.94)	5.55 (0.70)
Personal Control	5.53 (0.92)	5.31 (0.87)

**Table 2:** Descriptive statistics across experimental conditions in Study 2.

Variable	COVID-19 Condition Mean (*SD*)	Control Condition Mean (*SD*)
Social/Environmental Causes	4.81 (1.24)	4.76 (1.30)
Biological/Heredity Causes	3.96 (1.15)	4.25 (1.12)
Timeline	4.47 (0.82)	4.71 (0.88)
Treatability	5.56 (0.79)	5.68 (0.81)
Personal Control	5.53 (0.71)	5.38 (0.90)

## Data Availability

The data presented in this research are available on request from the corresponding author.

## Appendix A

### Appendix A.1. Study 1 Vignettes

#### Appendix A.1.1. COVID-19 Condition

Alex is 25 years old. During the COVID-19 pandemic, Alex began feeling unusually agitated for prolonged periods of time. Alex had a near-constant sense of worry about many different aspects of life. Often, Alex could not get to sleep due to fretting about a hypothetical risk to family or work life, which might never actually occur. The worry could spiral until Alex becomes sweaty, nauseous and light-headed. When this happened, Alex knew the fear was excessive and not logical, but Alex could not control it. Alex could not focus on anything and found even the simplest of tasks difficult to accomplish. This frustrated Alex’s work colleagues and meant that Alex was denied a promotion at work. In a conversation with Alex’s partner, Alex admitted to constantly feeling irritable and having a tendency to panic about minor setbacks or inconveniences. Alex’s partner thought that Alex may have been experiencing anxiety and encouraged Alex to see a doctor. Alex went to a doctor and was diagnosed with Generalised Anxiety Disorder.

#### Appendix A.1.2. Control Condition

Alex is a young adult. After turning 25, Alex began feeling unusually agitated for prolonged periods of time. Alex had a near-constant sense of worry about many different aspects of life. Often, Alex could not get to sleep due to fretting about a hypothetical risk to family or work life, which might never actually occur. The worry could spiral until Alex becomes sweaty, nauseous and light-headed. When this happened, Alex knew the fear was excessive and not logical, but Alex could not control it. Alex could not focus on anything and found even the simplest of tasks difficult to accomplish. This frustrated Alex’s work colleagues and meant that Alex was denied a promotion at work. In a conversation with Alex’s partner, Alex admitted to constantly feeling irritable and having a tendency to panic about minor setbacks or inconveniences. Alex’s partner thought that Alex may have been experiencing anxiety and encouraged Alex to see a doctor. Alex went to a doctor and was diagnosed with Generalised Anxiety Disorder.

## Appendix B

### Appendix B.1. Study 2 Vignettes

#### Appendix B.1.1. COVID-19 Condition

Alex is 25 years old. During the COVID-19 pandemic, Alex began feeling unusually low and sad for prolonged periods of time. Alex lost interest in old hobbies and found no enjoyment or pleasure in anything. Alex began eating less and lost weight. Alex felt tired throughout the day and had trouble sleeping at night. Alex withdrew from friends and found it hard to get the motivation to meet up with people or even reply to their messages. Alex couldn’t focus on anything and found even the simplest of tasks draining to accomplish. This frustrated Alex’s work colleagues and meant that Alex was denied a promotion at work. In a conversation with Alex’s partner, Alex expressed feeling hopeless about the future and believing that things would not improve. Alex’s partner thought that Alex may have been experiencing depression and encouraged Alex to see a doctor. Alex went to a doctor and was diagnosed with Major Depressive Disorder.

#### Appendix B.1.2. Control Condition

Alex is a young adult. After turning 25, Alex began feeling unusually low and sad for prolonged periods of time. Alex lost interest in old hobbies and found no enjoyment or pleasure in anything. Alex began eating less and lost weight. Alex felt tired throughout the day and had trouble sleeping at night. Alex withdrew from friends and found it hard to get the motivation to meet up with people or even reply to their messages. Alex couldn’t focus on anything and found even the simplest of tasks draining to accomplish. This frustrated Alex’s work colleagues and meant that Alex was denied a promotion at work. In a conversation with Alex’s partner, Alex expressed feeling hopeless about the future and believing that things would not improve. Alex’s partner thought that Alex may have been experiencing depression and encouraged Alex to see a doctor. Alex went to a doctor and was diagnosed with Major Depressive Disorder.
